# Detection of Arc/Arg3.1 oligomers in rat brain: constitutive and synaptic activity-evoked dimer expression *in vivo*

**DOI:** 10.3389/fnmol.2023.1142361

**Published:** 2023-06-09

**Authors:** Tadiwos F. Mergiya, Jens Edvard Trygstad Gundersen, Tambudzai Kanhema, Grant Brighter, Yuta Ishizuka, Clive R. Bramham

**Affiliations:** ^1^Department of Biomedicine, University of Bergen, Bergen, Norway; ^2^Mohn Research Center for the Brain, University of Bergen, Bergen, Norway

**Keywords:** Activity-regulated cytoskeleton-associated protein (Arc/Arg3.1), brain-derived neurotrophic factor (BDNF), dentate gyrus, *in situ* protein crosslinking, long-term potentiation (LTP), neuronal cell cultures, protein oligomerization, synaptic plasticity

## Abstract

The immediate early gene product activity-regulated cytoskeleton-associated protein (Arc or Arg3.1) is a major regulator of long-term synaptic plasticity with critical roles in postnatal cortical development and memory formation. However, the molecular basis of Arc function is undefined. Arc is a hub protein with interaction partners in the postsynaptic neuronal compartment and nucleus. Previous *in vitro* biochemical and biophysical analysis of purified recombinant Arc showed formation of low-order oligomers and larger particles including retrovirus-like capsids. Here, we provide evidence for naturally occurring Arc oligomers in the mammalian brain. Using *in situ* protein crosslinking to trap weak Arc–Arc interactions, we identified in various preparations a prominent Arc immunoreactive band on SDS-PAGE of molecular mass corresponding to a dimer. While putative trimers, tetramers and heavier Arc species were detected, they were of lower abundance. Stimulus-evoked induction of Arc expression and dimer formation was first demonstrated in SH-SY5Y neuroblastoma cells treated with the muscarinic cholinergic agonist, carbachol, and in primary cortical neuronal cultures treated with brain-derived neurotrophic factor (BDNF). In the dentate gyrus (DG) of adult anesthetized rats, induction of long-term potentiation (LTP) by high-frequency stimulation (HFS) of medial perforant synapses or by brief intrahippocampal infusion of BDNF led to a massive increase in Arc dimer expression. Arc immunoprecipitation of crosslinked DG tissue showed enhanced dimer expression during 4 h of LTP maintenance. Mass spectrometric proteomic analysis of immunoprecipitated, gel-excised bands corroborated detection of Arc dimer. Furthermore, Arc dimer was constitutively expressed in naïve cortical, hippocampal and DG tissue, with the lowest levels in the DG. Taken together the results implicate Arc dimer as the predominant low-oligomeric form in mammalian brain, exhibiting regional differences in its constitutive expression and enhanced synaptic activity-evoked expression in LTP.

## Introduction

Activity-regulated cytoskeleton-associated protein (Arc, also known as Arg3.1) is the product of a neuronal immediate early gene, with critical functions in synaptic plasticity, memory formation, and postnatal cortical maturation (reviewed in Bramham et al., 2010; Shepherd and Bear, 2011; Eriksen and Bramham, 2022). In activated glutamatergic neurons, Arc contributes to mechanisms of long-term potentiation (LTP) and long-term depression (LTD) of synaptic efficacy as well as homeostatic synaptic scaling (Guzowski et al., 2000; Plath et al., 2006; Shepherd et al., 2006; Messaoudi et al., 2007; Waung et al., 2008; Okuno et al., 2012; Wang et al., 2016). Biochemically, Arc is a protein-interaction hub with functions in the postsynaptic compartment and neuronal nucleus (Nikolaienko et al., 2018). Arc binding to partner proteins modulates trafficking of AMPA-type glutamate receptors (Chowdhury et al., 2006; DaSilva et al., 2016), actin cytoskeletal dynamics in dendritic spines (Messaoudi et al., 2007; Peebles et al., 2010; Nair et al., 2017), and contributes to regulation of chromatin state and transcription (Korb et al., 2013; Wee et al., 2014; Salery et al., 2016). However, it is not clear how Arc protein is targeted toward specific cellular functions (Zhang and Bramham, 2021).

Recombinant purified Arc can self-associate, forming stable low-order and higher-order oligomers, raising the possibility that Arc function is related to oligomeric state (Byers et al., 2015; Myrum et al., 2015; Eriksen et al., 2021). Arc evolved from ancient Ty3/Gypsy retrotransposons and has structural homology to HIV retroviruses (Campillos et al., 2006; Zhang et al., 2015). Mammalian Arc has two major domains separated by a disordered linker region (Myrum et al., 2015). The N-terminal domain (NTD) is a predicted anti-parallel coiled-coil (Hallin et al., 2018; Eriksen et al., 2021). The C-terminal domain (CTD), also known as the capsid (CA) domain, is a structural homolog of the retroviral Gag CA domain (Zhang et al., 2015). Recombinant Arc from mammals and *Drosophila* can self-assemble into virus-like capsid structures that harbor Arc mRNA (Ashley et al., 2018; Pastuzyn et al., 2018; Eriksen et al., 2021). Unveiling a new form of intercellular communication, evidence suggests that Arc capsids are released in extracellular vesicles and capable of delivering mRNA to neighboring cells (Ashley et al., 2018; Pastuzyn et al., 2018; Hantak et al., 2021).

Oligomerization of recombinant Arc is initiated by the NTD, with recent work demonstrating a critical role of coil-2 of the anti-parallel coiled-coil (Hallin et al., 2018; Eriksen et al., 2021). Mutation of a 7-amino acid oligomerization motif in coil-2 results in a dimer, suggesting the dimer is the building block for higher-order assembly (Eriksen et al., 2021). Retrovirus-like dimerization motifs in the CA domain are involved in assembly from tetramers to 32-mers (Zhang et al., 2019). A study of Arc knockin mice harboring mutations to the CA domain implicated Arc oligomerization in regulation of plasticity and learning (Zhang et al., 2019). *In vitro* biochemical studies suggest that Arc oligomeric state and capsid formation are regulated by Arc phosphorylation and interaction with protein ligands and mRNA (Pastuzyn et al., 2018; Nielsen et al., 2019; Zhang et al., 2019; Eriksen et al., 2021; Walczyk-Mooradally et al., 2021). Collating the available evidence, an oligomeric state hypothesis of synaptic plasticity was proposed, in which formation of stable Arc species dictates partner interactions and cellular functions (Eriksen and Bramham, 2022). However, evidence for Arc oligomers of any kind in the mammalian brain is lacking.

Here, we sought to detect endogenous Arc oligomers. Identification of homo-oligomers formed by weak non-covalent interactions is challenging due to disruption of interactions caused by cell lysis and sample processing. To stabilize interactions and facilitate capture of Arc complexes *in situ*, we applied cell permeable protein crosslinkers to live neuronal cell cultures and *ex vivo* to brain tissue samples following treatments that induce Arc expression. We identify the Arc dimer as the predominant low-oligomeric species. Enhanced expression of endogenous Arc dimer was observed following carbachol (Cch) treatment of SH-SY5Y neuroblastoma cells, BDNF treatment of cortical neuronal cultures, and LTP induction in the dentate gyrus (DG) of anesthetized rats, where increases of more than 20-fold were demonstrated. Arc immunoprecipitation and mass spectrometric proteomic analysis (IP-MS/MS) of gel-excised bands from DG samples corroborated detection of dimer. We also note region-specific difference in basal expression of dimer, with higher levels in the hippocampus compared to DG.

## Materials and methods

### Animals, electrophysiology, and tissue collection

*In vivo* electrophysiology experiments were conducted in adult male Sprague–Dawley rats weighing 250–500 g. All experimental procedures approved by Norwegian National Research Ethics Committee in compliance with EU Directive 2010/63/EU, ARRIVE guidelines. Experiments were conducted by Federation of Laboratory and Animal Science Associations (FELASA) C course-trained and certified researchers. Rats were anesthetized using urethane (1.5 g/kg, i.p.) and fixed on a stereotaxic frame as described in previous works. Briefly, medial perforant path fibers were stimulated using a bipolar electrode (NE-200, 0.5 mm tip separation, Rhodes Medical Instruments, Woodland Hills, CA, United States) placed in the angular bundle (7.9 mm posterior to the bregma, 4.2 mm lateral to the midline). Evoked field potentials were recorded by an insulated tungsten (0.075 mm; A-M Systems #7960) electrode placed in the dentate hilus (3.9 mm posterior to bregma, 2.3 mm lateral to midline). Test-pulse stimulation (0.033 Hz) was given during 20 min baseline recording before HFS and during post-HFS recording. HFS was delivered in three sessions of HFS separated by 5 min, with each session consisting of four, 400 Hz stimulus trains (8 pulses/train) with 10 s between trains. For BDNF-LTP induction, BDNF was infused directly into the stratum lacunosum molecular of CA1 using a cannula (31 gauge) with attached recording electrode (0.075 mm; A-M Systems #7960). The recording electrode was configured such that the tip of the cannula was ~700 μm above the hilar recording site. The other end of the cannula was connected, via a polyethylene (PE50) tube, to a Hamilton syringe (Reno, NV) mounted on an SP syringe pump (World Precision Instruments, Sarasota, FL). After a stable 20 min baseline recording, 1 μg of freshly prepared BDNF dissolved in PBS was infused over 15 min (60 nL/min). After the completion of recordings, rats were decapitated, and brains were taken out and dissected on ice. Ipsilateral (treated) and contralateral DG were rapidly and carefully micro-dissected on an ice-cold plate and frozen until further use.

Recorded signals from the dentate hilus were amplified, filtered (1 Hz to 10 kHz), and digitized (25 kHz). Evoked field potentials were acquired and analyzed using DataWave Technologies (Longmont, CO, United States) WorkBench software. The maximum fEPSP slope was measured from the leading positive peak. Four consecutive responses were averaged and plotted to show the time course of percentage changes in the fEPSP slope (relative to baseline). Student’s t-test was used for statistical analysis of baseline and post-HFS or BDNF-infusion recordings.

### Cell culture: transfection and pharmacological stimulation

Human embryonic kidney 293FT cells (HEK293FT, Thermo Fisher Scientific, Waltham, MA) were maintained in Dulbecco’s modified Eagle medium (DMEM/high-glucose, Sigma-Aldrich, St. Louis, MO, United States) supplied with 10% fetal bovine serum (FBS, Sigma-Aldrich) and 100 U/mL Penicillin-Streptomycin (Thermo Fisher Scientific). The cells were grown at 37°C in a humidified incubator with 5% CO_2_ to 80–90% confluency. For ectopic Arc expression, confluent HEK293FT cells were transfected with DNA plasmids expressing Arc N-terminally fused to monomeric Turquoise 2 (mTq2-Arc) or mTq2 alone under the CMV promoter. Briefly, plasmid DNAs were mixed (1:2 ratio) with Lipofectamine^™^ 2000 Transfection Reagent (Thermo Fisher Scientific) in Opti-MEM^™^/Reduced Serum Medium (Thermo Fisher Scientific 31985047) and incubated for 5 min at room temperature. Then, DNA-lipofectamine complexes were added dropwise to HEK293FT cells and incubated at 37°C in a humidified incubator with 5% CO_2_. A day later, an optimal expression of transfected genes was verified by mTq2 fluorescence maturation before cells were harvested and crosslinked.

Standard human SH-SY5Y neuroblastoma cells (ATCC® CRL-2266^™^ LGC Standards GmbH) and a stable SH-SY5Y line for Arc overexpression (Nikolaienko et al., 2017) were maintained in DMEM/high-glucose supplied with 10% FBS and 100 U/mL Penicillin–Streptomycin. The cells were grown at 37°C in a humidified incubator with 5% CO_2_ to 80–90% confluency. Wildtype SH-SY5Y cells were used to investigate intracellular Arc oligomerization dynamics following upregulation of endogenous Arc expression. Endogenous Arc expression in wildtype SH-SY5Y cells was induced by 100 μM Cch stimulation for 1 h at 37°C (Soulé et al., 2012). We also used SH-SY5Y cell lines generated for stable, constitutive expression of Arc. These SH-SYSY cells harbor a CMV::rArc-StrepII-HA cassette introduced by lentiviral particles (Nikolaienko et al., 2017). Stimulated neuroblastoma cells were then harvested, crosslinked and subjected to SDS-PAGE.

### Primary cortical neuronal culture

Primary cortical neuronal cultures were prepared and cryopreserved in the study of Ishizuka and Bramham (2020). Frozen neurons were thawed and plated as previously described (Ishizuka and Bramham, 2020). The cryovials were briefly placed in a 37°C water bath and gently mixed with 1 mL pre-warmed plating medium. Cell density of the suspension was adjusted by adding more plating medium to achieve 40,000–60,000 cells/cm^2^ on PLL-coated 6 cm culture dishes. After confirming complete attachment of neurons to the bottom of culture dishes (2–3 h after plating), the plating medium was changed to maintenance medium, composed of Neurobasal^™^ Medium (Thermo Fisher Scientific), 2% B-27™ supplement (Thermo Fisher Scientific), and 0.25% GlutaMAX^™^-I (Thermo Fisher Scientific). Glial proliferation was prevented by treating neuronal cultures with 1 μM cytosine arabinoside (AraC, Sigma-Aldrich) at 4 days *in vitro* (DIV). Neurons were maintained at 37°C in a humidified incubator with 5% CO_2_ by replacing 20% of old media with fresh media per week. At 21 DIV, neurons were stimulated with BDNF (50 ng/mL) for 2 h at 37°C and collected for *in situ* chemical crosslinking.

### Synaptoneurosome preparation

Synaptoneurosomes were isolated from hippocampal CA tissue following previously published methods with some modifications (Villasana et al., 2006; Ishizuka and Bramham, 2020). Briefly, both crosslinked and non-crosslinked CA tissue were homogenized by 10–12 gentle strokes in a Dounce homogenizer with a clearance of 0.1–0.15 mm (Thomas Scientific, Swedesboro, NJ, United States) in a synaptoneurosome buffer (pH 7.0), containing 10 mM Hepes, 1 mM EDTA, 2 mM EGTA, 0.5 mM DTT, and cOmplete^™^, EDTA-free Protease Inhibitor Cocktail (Sigma-Aldrich) at 4°C. A small sample of lysate was stored for immunoblot analysis while the remainder was processed for synaptoneurosomes. Lysates samples, kept ice-cold, were filtered twice through three layers of a prewetted 100 μm pore nylon net filter (Sigma-Aldrich) held in 13 mm diameter filter holders (Swinnex Filter Holder, Merck). Then, the filtrates were subjected to filtration through a pre-wetted 5 μm pore hydrophilic filter (Durapore^®^ Membrane Filter, Merck) held in 13 mm diameter filter holders. The filtrates were centrifuged at 1000 × *g* for 10 min. The supernatant, corresponding to a subcellular fraction containing cytoplasmic proteins not excluded by filtrations, was kept for immunoblotting. The pellet, corresponding to the synaptoneurosomal fraction, was resuspended in a buffer containing 0.32 M sucrose, and 1 mM NaHCO_3_ (pH 7.0).

### Chemical crosslinkers and *in situ* crosslinking

Homobifunctional, primary amine-reactive crosslinkers with different properties were used. These include disuccinimidyl glutarate (DSG, 326.26 g/mol, 7.7 Å spacer arm, Cat# 20593), disuccinimidyl sulfoxide (DSSO, 388.35 g/mol, 10.3 Å spacer arm, MS cleavable, Cat# A33545) and dithiobis (succinimidyl) propionate (DSP, 404.42 g/mol, 12 Å spacer arm, thiol-cleavable, Cat# 22586). All crosslinkers were obtained from Thermo Fisher Scientific and kept at 4°C in a moisture free container. Right before use, a 50 × (10 mM) fresh stock solution of crosslinker was prepared in DMSO.

*In situ* DSG crosslinking in HEK293FT cells was performed following methods described in Eriksen et al. (2021). Harvested cells were resuspended in ice-cold phosphate-buffered saline (PBS, pH 7.4) supplemented with cOmplete^™^, EDTA-free Protease Inhibitor Cocktail Tablet. Cell suspensions were then divided in two and incubated with 0.1 mM DSG or DMSO (equivalent volume or dilution) at 4°C for 10 min. For SH-SY5Y neuroblastoma cells and cortical neuronal cultures, we found crosslinking with 0.2 mM DSG for 10 min at 4°C to be optimal, using DJ-1 protein and β-actin as positive and negative controls for dimer formation, respectively. The crosslinking reaction was quenched with 50 mM Tris–HCl (pH 7.5) at room temperature for 15 min before cell lysis by sonication (40% maximum power for 15 s). Cell lysates were then centrifuged at 20,000 × *g* at 4°C for 15 min and the supernatant was collected. Protein levels were estimated using the Pierce BCA Protein Assay Kit (Thermo Fisher Scientific, Cat# 23227). Samples (equivalent to 30 μg proteins) were then subjected to SDS-PAGE separation.

Chemical crosslinking of tissue samples was performed according to previously described methods (Imberdis et al., 2019) with minor modifications. Briefly, frozen rat DG, CA, or cortical tissues were weighed, suspended in protease inhibitor containing PBS (pH 7.4), and placed flat on an ice-cold glass petri dish marked with ~ 0.5 mm grid spacing. Tissues were cut in ~0.5 mm slices and transferred to 1.5 mL Eppendorf tube. One ml of PBS was used for every 100 mg of wet tissue to achieve 100 mg/mL of chopped tissue suspensions. Tissue suspensions were then incubated with 0.5 mM crosslinker or DMSO solvent at 37°C for 30 min with shaking. This optimal crosslinking condition was achieved after a series of pilot experiments in which different final DSG concentrations (0.1, 0.2, 0.5, and 1 mM), incubation temperatures (4°C, room temperature and 37°C) and incubation times (10, 20 and 30 min) were tested. To prevent excessive crosslinking, 50 mM Tris–HCl (pH 7.5) was added and mixed with rocking at room temperature for 15 min. Total protein lysate was prepared by adding Triton X-100 (to 1% final concentration) and incubating for 30 min at 4°C before homogenization. Tissues were homogenized by 10–12 gentle strokes in a Dounce homogenizer with a clearance of 0.1–0.15 mm. The homogenates were then centrifuged (10,000 × *g*, 10 min at 4°C) to collect the supernatant and protein concentration was estimated using BCA protein assay before downstream biochemical experiments.

### Antibodies

Primary antibodies used in this study include mouse monoclonal anti-Arc (C-7; Santa Cruz Biotechnology, Dallas, TX, Cat# sc-17839, RRID:AB_626696), rabbit polyclonal anti-Arc (Synaptic Systems, Göettingen, Germany, Cat# 156003, RRID:AB_887694), mouse anti-DJ-1 (Santa Cruz Biotechnology Cat# sc-55572, RRID:AB_831639), mouse anti-β-tubulin (Santa Cruz Biotechnology Cat# sc-166729, RRID:AB_2010699) and mouse anti-β-actin (Sigma-Aldrich Cat# A5441, RRID:AB_476744). Specificity of the anti-Arc antibodies has been demonstrated by Arc knockout and knockdown (Gao et al., 2018, 2019; Leung et al., 2022). Secondary antibodies are as follows: goat anti-mouse IgG, H & L chain peroxidase conjugated antibody (Merck Cat# 401253, RRID:AB_437779) and goat anti-rabbit IgG, H & L chain peroxidase conjugated antibody (Merck Cat# 401315, RRID:AB_2617117). Secondary antibodies for detecting immunoprecipitated proteins are peroxidase affinipure goat anti-mouse IgG, light chain specific (Jackson ImmunoResearch Labs, West Grove, PA, Cat# 115–035-174, RRID:AB_2338512) and peroxidase IgG Fraction Monoclonal Mouse Anti-Rabbit IgG, light chain specific (Jackson ImmunoResearch Labs, Cat# 211–032-171, RRID:AB_2339149). We also used ALFA-epitope tagged anti-Arc nanobody (Nb) H11 for immunoprecipitation (generated by NanoTag Biotechnologies, Göttingen, Germany). ALFA-H11 specifically binds the Arc N-lobe and is suitable for immunopurification of Arc from cell lines and tissue samples (Ishizuka et al., 2022; Markússon et al., 2022).

### Immunoprecipitation

Lysates of DSG crosslinked tissue samples were incubated with rabbit anti-Arc antibody or ALFA-H11 anti-Arc nanobody. Briefly, 20 μL of Pierce™ Protein A/G (Thermo Fisher Scientific, Cat# 20421) agarose beads or ALFA Selector^ST^ (for “Super Tight”) resin (Götzke et al., 2019; NanoTag Biotechnologies, Cat# N1511) were washed twice and resuspended in PBST (PBS containing 0.01% Tween-20 and protease inhibitor, pH 7.4). Protein A/G agarose beads were then mixed with 2 μg of anti-Arc antibody and incubated with rocking at room temperature for 1 h. 2 μg ALFA-H11 was adsorbed onto the ALFA Selector^ST^ resins by rocking for 1 h at 4°C. Then, lysates were added and incubated with anti-Arc antibody/beads or ALFA-H11/ALFA Selector^ST^ resin mix overnight at 4°C with head-over-tail rotation. Protein A/G agarose beads and ALFA Selector^ST^ resins were washed three times with PBST. The immunoprecipitate was then eluted by boiling in 2X Laemmli sample buffer (Bio-Rad Laboratories, Inc., Hercules, CA, United States) containing 100 mM DTT and separated on SDS-PAGE.

### Mass spectrometry

Immunoprecipitated samples were separated on SDS-PAGE and stained in-gel with Coomassie brilliant blue (CBB) to visualize discrete bands of higher molecular mass (M_r_) matching identified Arc immunoreactive bands of interest. Excised gels were then in-gel digested, and peptides were separated using electrospray liquid chromatography–tandem mass spectrometry (LC–MS/MS) as described previously (Myrum et al., 2017; Cappelletti et al., 2022). About 0.5 μg protein as tryptic peptides dissolved in 2% acetonitrile (ACN)/0.5% formic acid (FA), were injected into an Ultimate 3000 RSLC system (Thermo Scientific, Sunnyvale, CA, United States) connected online to an Exploris 480 mass spectrometer (Thermo Scientific, Bremen, Germany) equipped with EASY-spray nano-electrospray ion source (Thermo Scientific). Samples were loaded and desalted on a pre-column (Acclaim PepMap 100, 2 cm × 75 μm ID nanoViper column, packed with 3 μm C18 beads, Thermo Fisher Scientific, Carlsbad, CA, United States) with 0.1% trifluoroacetic acid (TFA, *v*/*v*) at a flow rate of 5 μL/min for 5 min. Then, peptides were separated during a biphasic ACN gradient from two nanoflow UPLC pumps (flow rate of 250 nL/min) on a 25 cm analytical column (PepMap RSLC, 25 cm × 75 μm ID EASY-spray column, packed with 2 μm C18 beads). Solvents A and B were 0.1% FA (*v*/*v*) in water and 100% ACN, respectively. The gradient composition was 5% B during trapping (5 min) followed by 5–6% B over 1.5 min, 6–24% B for the next 88.5 min, 24–32% B over 15 min, and 32–85% B over 2 min. Elution of very hydrophobic peptides and conditioning of the column were performed during 10 min isocratic elution with 85% B and 15 min isocratic elution with 5% B, respectively. The instrument was controlled through Thermo Scientific SII for Xcalibur 1.6.

Peptides eluted from the column were detected in the Exploris 480 Mass Spectrometer with high field asymmetric waveform ion mobility spectrometry (FAIMS) enabled and “Advanced Peak Determination” on. FAIMS was enabled using two compensation voltages (CVs), − 45 V and − 65 V, respectively. During each CV, the mass spectrometer was operated in the data-dependent-acquisition (DDA)-mode to automatically switch between one full scan MS and MS/MS acquisition controlled via Orbitrap Exploris 480 Tune 3.1 and Xcalibur 4.4. The cycle time was maintained at 1.5 s/CV. MS spectra were acquired in the scan range 375–1,500 m/z with a resolution of 120,000 at m/z 200, automatic gain control (AGC) target of 3e6 and a maximum injection time (IT) at auto (depending on transient length in the orbitrap). The most intense eluting peptides with charge states 2 to 5 were sequentially isolated to a standard target value (AGC, 1e5) and a maximum IT of 75 ms in the C-trap, and isolation width maintained at 1.6 m/z (quadrupole isolation), before fragmentation in the higher energy collision dissociation. Fragmentation was performed with a normalized collision energy of 30%, and fragments were detected in the Orbitrap at a resolution of 15,000 at m/z 200, with the first mass fixed at m/z 110. One MS/MS spectrum of a precursor mass was allowed before dynamic exclusion for 30 s with “exclude isotopes” on. Lock-mass internal calibration was not enabled. Ion spray voltage of 1900 V, no sheath and auxiliary gas flow, and capillary temperature of 275°C were set as spray and ion-source parameters.

The Proteome Discoverer^™^ software (version 2.5, Thermo Fisher Scientific, Carlsbad, CA, United States) was used to analyze the raw files from the LC–MS/MS. Peak lists were searched against the rat SwissProt FASTA database (version November 2022), and common contaminants database by Sequest HT. Methionine oxidation/acetylation on protein N-terminus and cysteine carbamidomethylation were included as variable and fixed modifications, respectively. For proteins and peptides, the false discovery rate was set to 0.01. The list of identified proteins was exported to Microsoft^®^ Excel^®^ (Version 2210 Build 16.0.15726.20188) for analysis. The normalized Arc abundances were log2 transformed and plotted on Graphpad prism (version 9.4.1 for Windows, GraphPad Software, San Diego, CA, United States).

### Electrophoresis and immunoblotting

SDS-PAGE was performed as previously described (Ishizuka and Bramham, 2020; Ishizuka et al., 2022). Non-crosslinked and crosslinked samples from cell lines, primary neurons and brain tissues were mixed with 4× Laemmli sample buffer (Bio-Rad Laboratories, Inc.) supplemented with 200 mM DTT and denatured by boiling at 95°C for 5 min. Immunoprecipitated complexes were first eluted using 2× Laemmli sample buffer containing 100 mM DTT before denaturation. Proteins/protein complexes were then separated on a homemade 7.5% Tris–HCl gel by electrophoresis. Separated proteins were blotted to Nitrocellulose membrane (Bio-Rad Laboratories, #1620112) and transfer efficiency was checked by a brief Ponceau S incubation. Membranes were then blocked with 5% dry non-fat milk powder in Tris-buffered saline with 0.05% Tween-20 (TBST) at room temperature for 1 h before overnight incubation with primary antibodies. Then, membranes were incubated with appropriate peroxidase conjugated secondary antibodies and probed for peroxidase activity using Clarity Western ECL substrate (Bio-Rad Laboratories, #1705061). Generated chemiluminescence was then imaged using Image Lab^™^ Software (Gel Doc^™^ XR +, Bio-Rad Laboratories). Densitometry analysis of images was performed using ImageJ/FIJI (RRID: RRID:SCR_002285). All densitometric quantifications of dimer and monomer were made using images without saturated pixels using exposure times within the linear range of detection. For direction visual comparison of Arc species some of blots shown have saturated pixels due to high expression on total monomer in non-crosslinked samples. Optical densities were exported to Microsoft^®^ Excel^®^ and analyzed using Graphpad prism (statistics details for different samples are given in Supplementary Table 1).

## Results

### Identification of putative endogenous Arc dimers and low-order oligomers by *in situ* crosslinking in neuroblastoma SH-SY5Y cells

**Figure 1:**
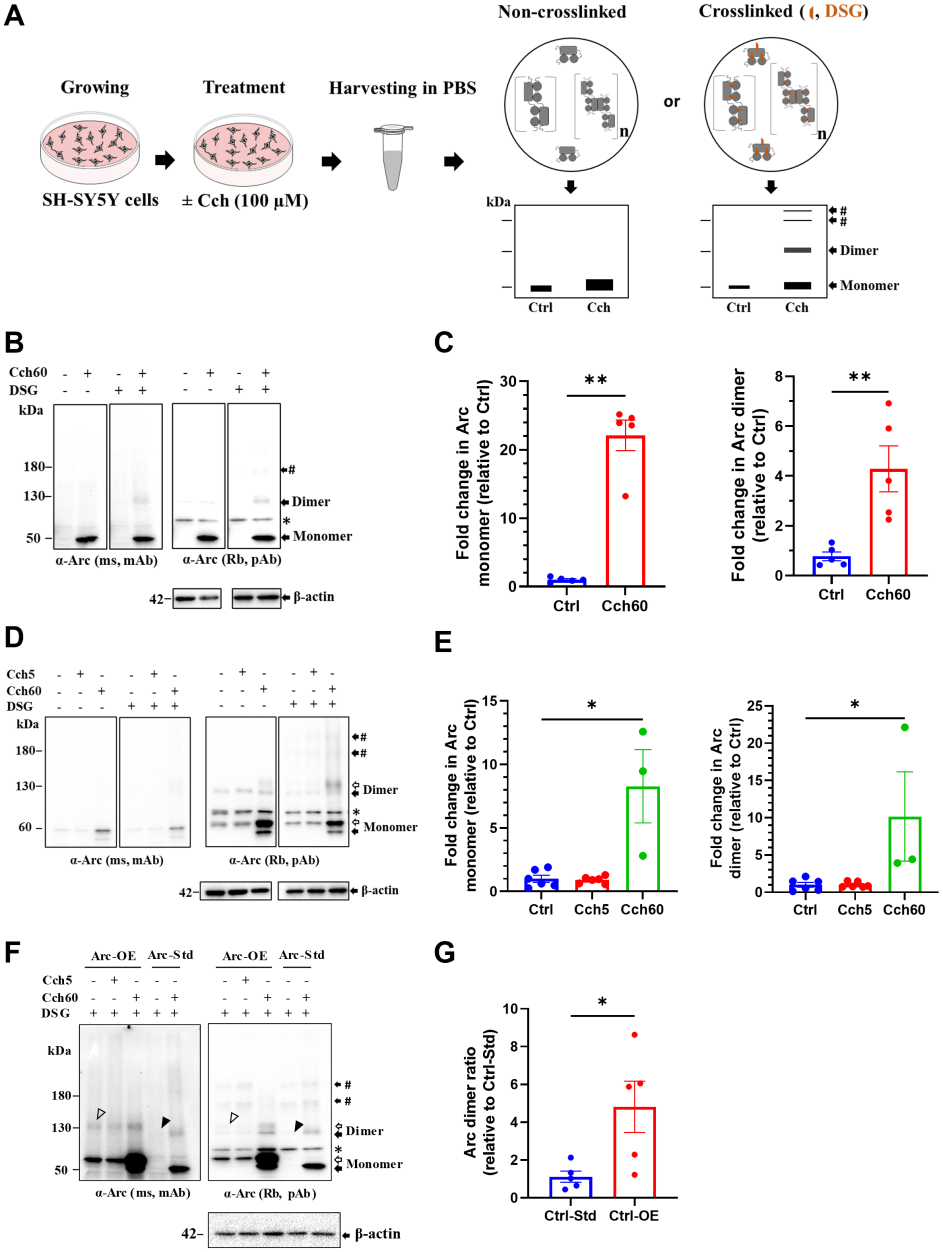
Putative Arc dimers and low-order oligomers were detected in SH-SY5Y cells. **(A)** Schematics of *in situ* disuccinimidyl glutarate (DSG) crosslinking of SH-SY5Y cells. Prediction of preserved Arc species by crosslinking depicted on SDS-PAGE cartoons. **(B)** Immunoblot analysis of Arc species in standard SH-SY5Y cells. After SDS-PAGE, membranes were probed with monoclonal mouse (top left) and polyclonal rabbit (top right) anti-Arc antibodies and anti-β-actin (bottom). Black arrows refer to specific Arc immunoreactive bands corresponding to monomer (50 kDa) and putative dimer at 130 kDa. The # sign indicates a low-order oligomer larger than the dimer. The asterisk refers to a non-specific band. **(C)** Densitometric quantification of immunoblots for Cch treated SH-SY5Y cells showing fold change in Arc monomer (left panel) and dimer (right panel) relative to control. Analysis of total Arc monomer is based on non-crosslinked samples. Values are expressed as mean ± standard error of the mean (SEM; *n* = 5). Mann–Whitney *U* test, ^**^p < 0.01. **(D)** Representative Arc immunoblots in Arc-StrepII-HA overexpressing SH-SY5Y cells. Black and open arrows show endogenous and exogenous Arc immunoreactive bands, respectively. # sign indicates low-order oligomers larger than dimers. The asterisk refers to a non-specific band. **(E)** Densitometric quantification showing fold change in Arc monomer (left panel) and dimer (right panel) relative to control. Values are expressed as mean ± SEM (*n* = 6 for Ctrl and Cch5, *n* = 3 for Cch60). ^*^p < 0.05. Kruskal–Wallis test with Dunn’s multiple comparisons. **(F)** Immunoblot showing Arc species in Arc overexpressing (Ctrl-OE) SH-SY5Y cells and standard (Ctrl-Std) SH-SY5Y cells. Putative Arc dimers in unstimulated Ctrl-OE and Ctrl-Std are shown with open and closed arrowheads, respectively. **(G)** Fold change in Arc dimer ratio in unstimulated Arc overexpressing (Ctrl-OE) SH-SY5Y cells relative to unstimulated standard (Ctrl-Std) SH-SY5Y cells. Values are expressed as mean ± SEM (*n* = 5). Mann–Whitney *U* test, ^*^p < 0.05.

We first used human neuroblastoma SH-SY5Y cells to probe for endogenous Arc monomers and higher M_r_ complexes by immunoblotting. As in previous studies (Soulé et al., 2012), Arc expression was induced by treatment with a muscarinic cholinergic agonist, carbachol (Cch) for 60 min (Figure 1A). In Cch treated, non-crosslinked cultures, Arc immunoblotting of samples separated on SDS-PAGE showed a prominent ~ 50 kDa monomer band and a weaker, discrete band at 120–130 kDa (hereafter referred to as ~ 130 kDa). Cch induced a 22-fold increase in Arc monomer levels relative to untreated control cultures (Figures 1B,C). Changes in monomer expression here and elsewhere in this study were assessed in non-crosslinked, DMSO treated cells. With *in situ* DSG crosslinking, the ~ 130 kDa band and a heavier Arc-immunoreactive band (> 180 kDa, indicated by #) were effectively trapped (Figure 1B). Immunoblot detection of the 130 kDa band was corroborated with mouse monoclonal and rabbit polyclonal anti-Arc antibodies (Figure 1B). Based on the M_r_ and band patterns, we refer to the 130 kDa Arc species as a putative dimer while discrete heavier bands are consistent with trimers or tetramers. The rabbit polyclonal antibody showed higher sensitivity and clearly resolved heavier bands (Figure 1B, right panel). We therefore based densitometric analysis on use of the rabbit polyclonal antibody. Cch treatment resulted in a 4-fold significant increase in 130 kDa Arc dimer band compared to untreated cells, using β-actin as a loading control for normalization (Figure 1C, right panel).

To enhance detection of oligomers, we also used neuroblastoma SH-SY5Y cells engineered for stable expression of Arc. These cells harbor a CMV::rArc-StrepII-HA cassette, introduced by lentiviral particles, in addition to the endogenous Arc gene (Nikolaienko et al., 2017). This cell line for constitutive Arc expression was previously used to study Cch-induced Arc phosphorylation (Nikolaienko et al., 2017). Under basal conditions, SH-SY5Y cells primarily expressed exogenous Arc in which monomers were detected at ~ 60 kDa (Figure 1D, open arrow). There was no effect of 5 min Cch stimulation on the Arc expression pattern. However, 60 min Cch stimulation (Cch60), significantly increased both exogenous and endogenous Arc expression, evidenced by prominent ~ 60 kDa (open arrow) and ~ 50 kDa (closed arrow) monomer bands, respectively (Figures 1D,E). Without crosslinking, apparent dimers of endogenous Arc and tagged-Arc (~130 kDa) were detected in untreated cells as well as Cch5 and Cch60 stimulated cells, but no heavier (> 180 kDa) Arc immunoreactive complexes were detected. With DSG crosslinking, discrete bands larger than 180 kDa appeared, indicating trapping of heavier Arc species (Figure 1D). Cch treatment for 60 min elicited a 10-fold increase in Arc dimer relative to non-treated cells (Figure 1E). Arc dimer levels in unstimulated, stably expressing cells were significantly increased 5-fold above levels in standard SH-SY5Y cells (Figures 1F,G), indicating constitutive dimerization during CMV promoter-driven Arc expression.

As a positive control for validation of crosslinking, we immunoblotted for DJ-1, an oxidative stress sensor protein. DJ-1 exists as a dimer (46 kDa) and monomer (23 kDa; Dettmer et al., 2013). Under optimal crosslinking conditions, fractions of naturally existing DJ-1 dimers are trapped and detected along with free monomer (Dettmer et al., 2013). As shown in Supplementary Figure S1A (left panel), our crosslinking conditions resulted in reliable detection of DJ-1 dimers, whereas only monomers were detected without DSG treatment. As a control for non-specific, excessive crosslinking, we immunoblotted for the monomeric protein, β-tubulin. β-tubulin was detected exclusively as a monomer in DSG crosslinked samples, indicating a lack of excessive crosslinking (Supplementary Figure S1A, right panel).

### Brain-derived neurotrophic factor induces Arc dimer formation in primary cortical neuronal cultures

**Figure 2:**
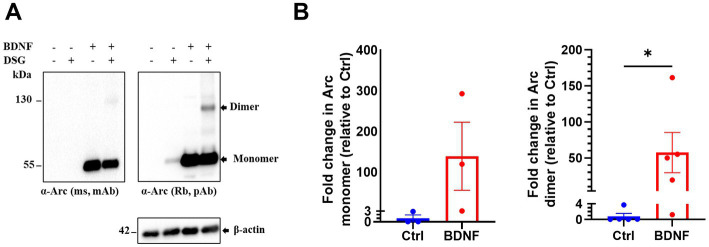
Brain-derived neurotrophic factor (BDNF) induces endogenous Arc dimer formation in primary cortical neuronal cultures. **(A)** Immunoblot analysis of Arc species *in situ* crosslinked, BDNF treated primary cortical neurons. Blotted proteins were immunoprobed using a monoclonal mouse (top left) and a polyclonal rabbit (top right) anti-Arc antibodies and anti-β-actin (bottom). **(B)** Densitometric quantification of fold change in Arc monomer expression (left) and dimer (right) relative to control. Values are expressed as mean ± SEM (*n* = 3 for monomer and 5 for dimer). Mann–Whitney *U* test. ^*^p < 0.05.

Next, we investigated endogenous Arc oligomerization in BDNF treated primary cortical neuronal cultures. In control untreated neurons, Arc was barely detectable by immunoblotting. BDNF treatment increased Arc monomer expression (Figures 2A,B) in both non-crosslinked and crosslinked cortical neurons. In crosslinked cultures, the 130 kDa Arc immunoreactive band was prominent only in BDNF treated samples. Densitometry showed a 58-fold increase in the Arc dimer band relative to unstimulated, crosslinked neurons (Figure 2B, right panel). Thus, BDNF stimulation of cortical neurons increases Arc expression and formation of Arc dimers as captured by *in situ* DSG crosslinking.

### Expression of putative Arc dimer after LTP induction in rat DG *in vivo*

**Figure 3:**
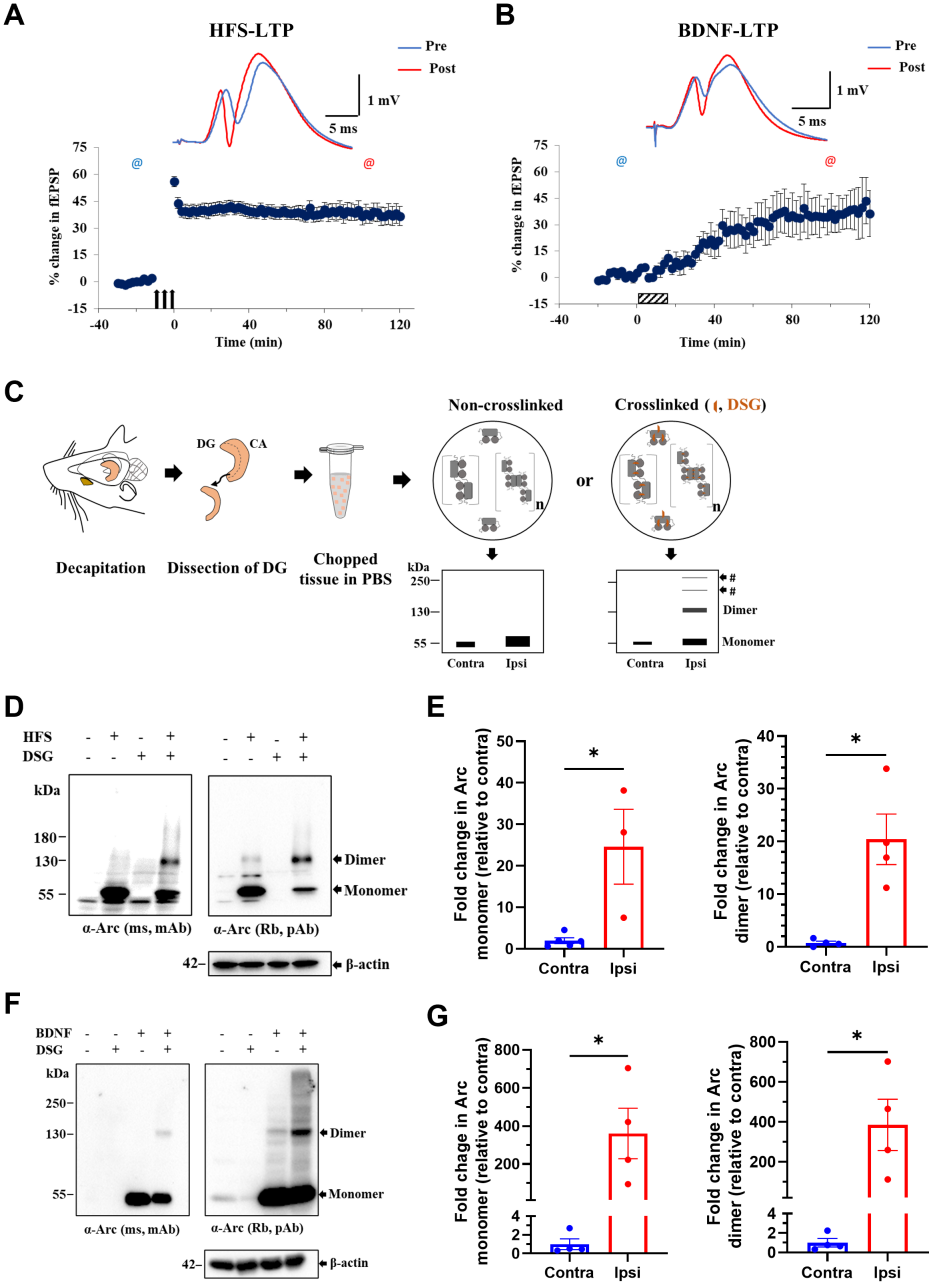
Enhanced expression of putative Arc dimer following HFS-LTP and BDNF-LTP in rat dentate gyrus (DG) *in vivo*. **(A)** HFS-induced LTP in anesthetized rats. Time course plots of percent change in fEPSP slope post-HFS relative to baseline. Values are mean ± SEM. Filled arrows represent three sessions of HFS. Top panel shows sample field potentials recorded pre-HFS (blue) and post-HFS (red) at timepoints indicated by @ signs. **(B)** BDNF-induced LTP. Hatched box indicates period of BDNF infusion. Sample field potentials recorded pre (blue) and post (red) BDNF at timepoints indicated by @ sign. **(C)** Schema of DSG *in situ* crosslinking in rat dentate tissues. Prediction of preserved Arc species by crosslinking was depicted on SDS-PAGE cartoons. **(D)** Immunoblot analysis of DSG crosslinked, HFS treated DG tissues. Blotted proteins were immunoprobed using monoclonal mouse (top left) and polyclonal rabbit (top right) anti-Arc antibodies and anti-β-actin (bottom). **(E)** Densitometric quantification of immunoblots for HFS-LTP. Left: fold change in Arc monomer expression relative to contralateral DG. Right: fold change in Arc dimer relative to contralateral DG. Values are expressed as mean ± SEM (*n* = 3–5). Mann–Whitney test, ^*^p < 0.05. **(F)** Immunoblot analysis of DSG crosslinked, BDNF treated DG tissues. Blotted proteins were immunoprobed using a monoclonal mouse (top left) and a polyclonal rabbit (top right) anti-Arc antibodies and anti-β-actin (bottom). **(G)** Densitometric quantification of immunoblots for BDNF-LTP. Left: fold change in Arc monomer expression relative to contralateral DG. Right: fold change in Arc dimer relative to contralateral DG. Values are expressed as mean ± SEM (*n* = 4). Mann–Whitney, ^*^p < 0.05.

Next, we applied DSG crosslinking to examine Arc immunoreactive species during synaptic plasticity *in vivo*. HFS-LTP in the DG is associated with robust Arc transcription in dentate granule cells, delivery of mRNA to dendrites, and sustained Arc synthesis critical for LTP consolidation (Steward et al., 1998; Messaoudi et al., 2007; Panja et al., 2014). We used 400 Hz burst stimulation to induce stable LTP at medial perforant-DG synapses in anesthetized rats (Figure 3A). Ipsilateral, HFS treated and contralateral, non-stimulated DG were cut into 500 μm-thick pieces and exposed to DSG or DMSO only (Figure 3C). A 25-fold increase in ~ 55 kDa Arc monomer was detected in ipsilateral DG compared to contralateral DG, confirming activity-evoked upregulation (Figures 3D,E). In non-crosslinked samples, a weak ~ 130 kDa putative dimer band was also detected in HFS treated but not contralateral control DG. With crosslinking, the 130 kDa dimer band became prominent, as the monomer band in the same lane was reduced relative to non-crosslinked sample (Figures 3D,E). Arc dimer expression was significantly increased 20-fold in ipsilateral DG compared to contralateral control (Figure 3E, right panel). The efficacy of crosslinking was again validated by the detection of endogenous DJ-1 dimers in crosslinked but not non-crosslinked tissue (Supplementary Figure S1B).

Exogenous application of BDNF triggers a slowly developing, protein synthesis-dependent potentiation termed BDNF-LTP (Kang and Schuman, 1996; Messaoudi et al., 1998, 2002; Ying et al., 2002). Unlike HFS-LTP, BDNF-LTP does not require NMDA receptor activation (Messaoudi et al., 2002). However, both the induction and consolidation of BDNF-LTP require Arc synthesis (Messaoudi et al., 2007; Kuipers et al., 2016). We therefore asked whether BDNF infusion is sufficient to induce Arc oligomerization. Intrahippocampal infusion of BDNF (1 μg, 15 min) triggered LTP that plateaued at 2 h post-infusion (Figure 3B) and resulted in a 350-fold upregulation in Arc monomer expression (Figures 3F,G). In non-crosslinked samples, only Arc monomers were detected in ipsilateral DG. In contrast, in crosslinked samples, BDNF-LTP was accompanied by a 385-fold increase in Arc dimer expression (Figure 3G, right panel).

We also assessed the relative levels of Arc dimer to monomer as detected in the same lane of DSG crosslinked samples. There was no significant difference between HFS and BDNF in the mean ratio of dimer:monomer (HFS: 0.40 ± 0.21; BDNF: 0.56 ± 0.24, at 2 h post-treatment Supplementary Figure S2).

### Enhanced expression of immunoprecipitated, 130 kDa Arc during HFS-LTP maintenance

**Figure 4:**
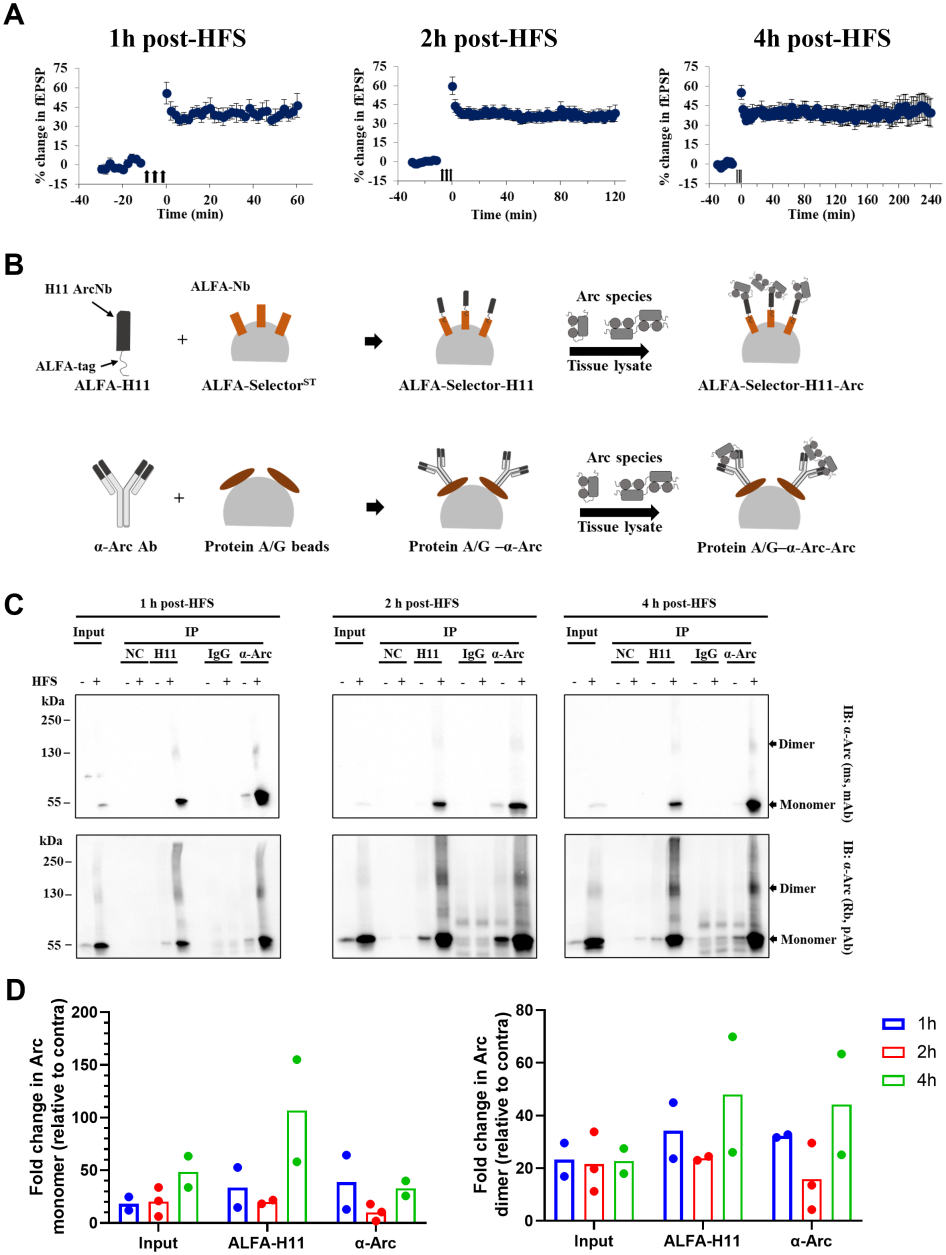
Stable enhanced expression of immunoprecipitated Arc dimer during HFS-LTP maintenance in rat DG *in vivo*. **(A)** Time course of percent change in fEPSP slope relative to the baseline plotted for 1, 2, and 4 h DG LTP. Values are mean ± SEM. Filled arrows represent three sessions of HFS. **(B)** Schema of immunoprecipitation of Arc species from pooled and crosslinked DG using anti-Arc antibody and ALFA-H11 nanobody. **(C)** Immunoblot analysis of purified immunocomplexes. Following SDS-PAGE, whole lysate (input) and Arc immunoprecipitated samples were blotted and probed with monoclonal mouse (top panel) and polyclonal rabbit (bottom panel) anti-Arc antibodies. **(D)** Densitometric quantification of immunoblots. Left: fold change in Arc monomer expression relative to contralateral DG. Right: fold change in Arc dimer relative to contralateral DG. Values are expressed as mean (*n* = 2 for 1 and 4 h post-HFS and *n* = 3 for 2 h post-HFS). DG from four rats were pooled for each observation.

To assess dynamics of Arc dimer expression during the first hours of LTP maintenance, DG tissue was collected at 1, 2 and 4 h post-HFS (Figure 4A), and immunoprecipitation was performed using pooled samples (DG from 4 rats) to enrich for Arc complexes (Figure 4B). As shown in Figure 4C, we confirmed enrichment of Arc monomer (~ 55 kDa) and putative dimer (~ 130 kDa) in the ipsilateral HFS treated DG using anti-Arc antibody or anti-Arc nanobody (ALFA-H11) for immunoprecipitation and both monoclonal and polyclonal antibodies for detection. Confirming specific immunoprecipitation, these bands were absent in samples incubated with ALFA-selector only or IgG-conjugated beads (Figure 4C). Immunoblot analysis showed enhanced expression of the 130 kDa band in immunoprecipitated samples and corresponding input samples at all time points. Arc dimer levels in the input sample were stably elevated ~ 22-fold in ipsilateral DG relative to contralateral control (Figure 4D, right panel, input), with more variability in fold-change observed in immunoprecipitated samples. In addition, there was an increase in Arc immunoreactivity across the higher M_r_ range appearing as a smear (Figure 4C).

### Prominent constitutive Arc dimer in hippocampus and cortex but not DG

**Figure 5:**
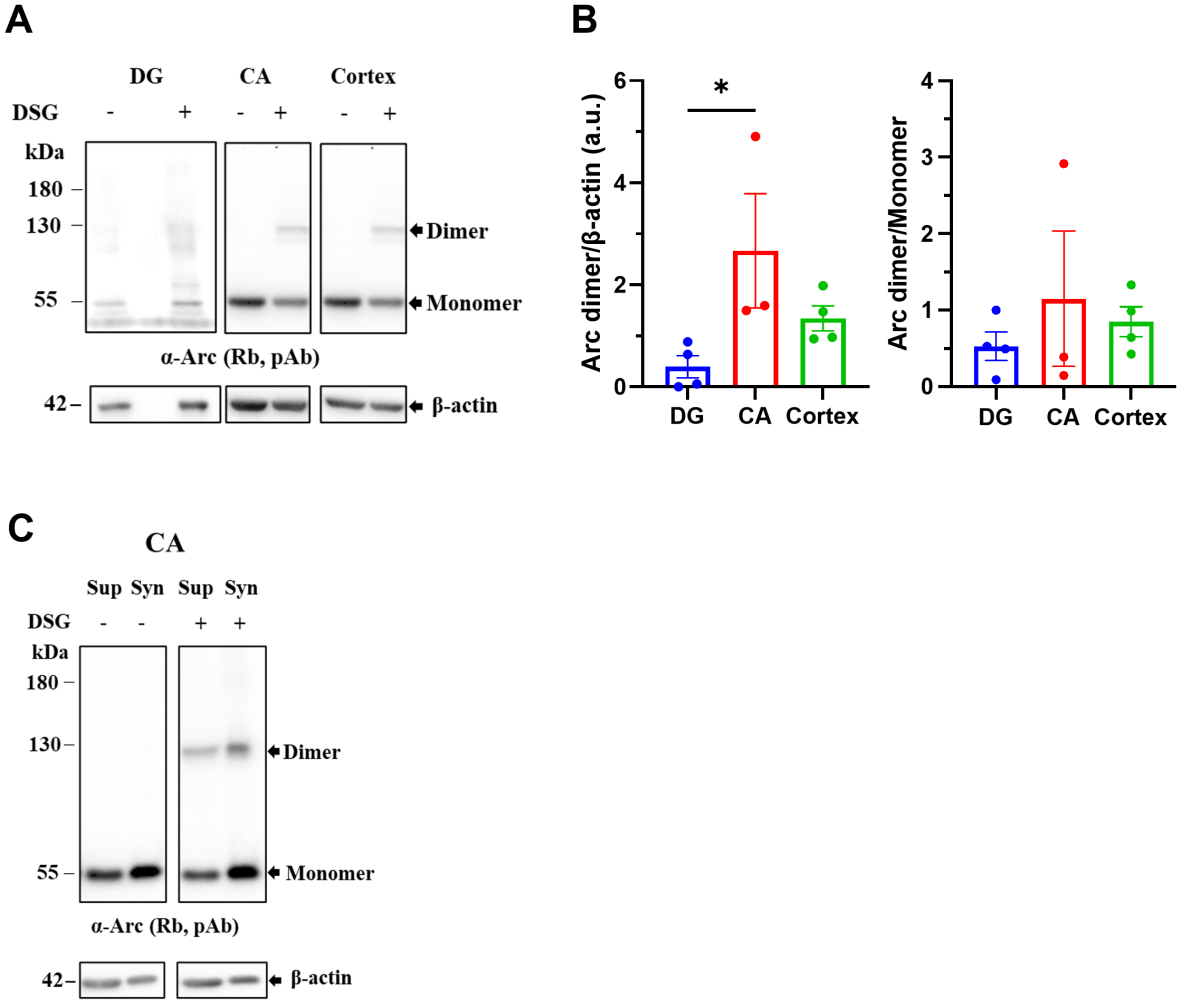
Prominent basal expression of Arc dimer in hippocampus and cortex but not DG. **(A)** Immunoblots from dentate gyrus (DG), hippocampal cornu ammonis (CA) and cortical tissue of naive, untreated rats. Tissues were crosslinked and separated on SDS-PAGE and membranes were probed with polyclonal rabbit anti-Arc antibodies (top) and anti-β-actin (bottom). **(B)** Quantification of Arc dimer expression normalized to β-actin (left) and dimer/monomer ratio in the DSG lane in DG, CA and cortex (right). Values are expressed as mean ± SEM (*n* = 3) for CA, *n* = 4 for DG and cortex. Kruskal-Wallis test with Dunn’s multiple comparisons test, ^*^p < 0.05. **(C)** Distribution of Arc dimer in supernatant (Sup) and synaptoneurosome (Syn) fractions isolated from crosslinked CA tissue.

The function of basal, constitutive Arc as detected without experimental treatments to induce transcription and translation, is little known. In non-stimulated DG, we detected Arc monomer but only negligible levels of Arc dimer under the crosslinking conditions used (Figure 5A). However, we considered that constitutive Arc formation may differ between regions. To this end we collected the hippocampal cornu ammonis (CA) region and cortex from naïve rats In the absence of crosslinking, only Arc monomers were detected in CA and cortex. Crosslinking revealed a prominent dimer band in CA and cortical samples (Figure 5A), and densitometric analysis showed a significantly higher expression of dimer in CA relative to DG (Figure 5B, left panel). However, there was no significant difference in the dimer:monomer ratio as measured in the DSG lane, indicating a similar proportion of dimer expression across these brain regions (Figure 5B, right panel). CA lysates were also fractionated to derive synaptoneurosomes. The ~ 130 kDa Arc was detected in synaptoneurosome and enriched ~ 2-fold relative to the supernatant (cytosolic) fraction (Figure 5C).

We also assessed DSG crosslinking in naïve DG, CA and cortical tissues by immunoblotting for DJ-1 and β-tubulin. We detected DJ-1 dimers in addition to monomers, and monomeric β-tubulin confirming optimal crosslinking (Supplementary Figure S1C). To further validate the crosslinking data, CA tissue was treated with the cleavable crosslinker dithiobis (succinimidyl) propionate (DSP). The thiol containing reducing agent, DTT, breaks the disulfide bond contained within the DSP arm to reverse the crosslinking. Crosslinked samples were denatured in sample buffer with DTT (reducing SDS-PAGE) or without DTT (non-reducing SDS-PAGE) and probed with anti-Arc antibodies. In non-reducing SDS-PAGE, both DSG and DSP effectively trapped Arc dimers and larger oligomers (Supplementary Figure S3A, right panel, shown by #). Putative Arc dimers and oligomers were lost under reducing SDS-PAGE in DSP, but not DSG, crosslinked samples (Supplementary Figure S3A, left panel). Thus, DSP treatment allows reversible crosslinking of the Arc complexes. This was also shown for trapping of DJ-I dimers as a positive control (Supplementary Figure S3B).

### Mass spectrometric proteomic validation of enhanced endogenous Arc dimer expression in LTP *in vivo*

**Figure 6:**
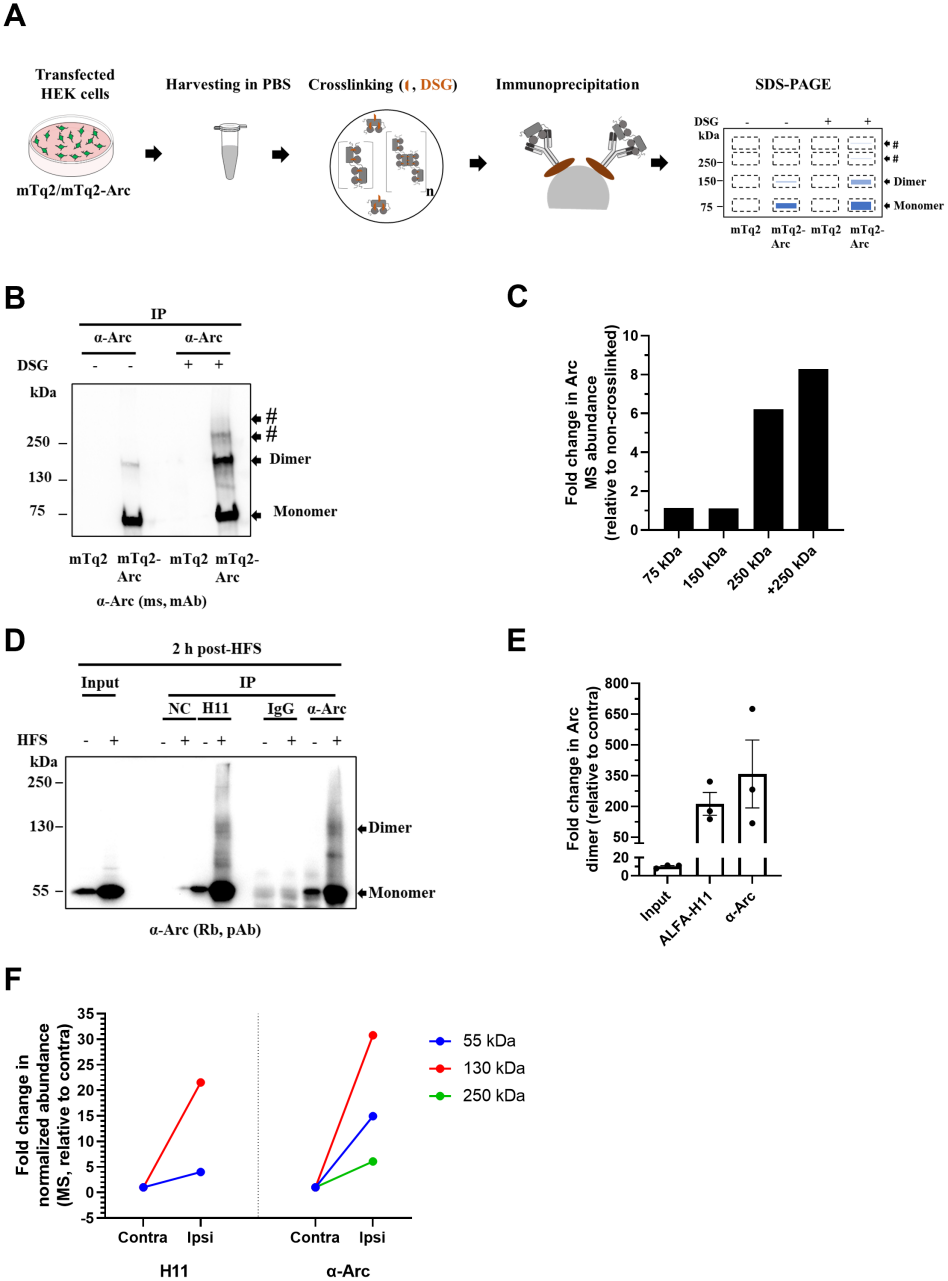
Mass spectrometry validation of heavier Arc species in HEK293FT cells *in vitro* and DG LTP *in vivo*. **(A)** Schematics of *in situ* DSG crosslinking of mTq2-Arc transfected HEK293FT cells and sample preparation for MS. Immunoprecipitated complexes were separated on SDS-PAGE and bands of interest (~75, 150, 250 and > 250 kDa, shown by broken rectangles) were visualized by Coomassie brilliant blue (CBB) gel staining, excised and processed for MS proteomic analysis. **(B)** Representative immunoblot of immunoprecipitated oligomers from mTq2-Arc expressing HEK293FT cells. Membranes were probed with mouse anti-Arc antibody to identify immunoreactive bands of interest. **(C)** Verification of Arc presence in putative oligomer bands. Fold change in normalized Arc abundance (MS) relative to non-crosslinked HEK293FT cells. **(D)** Representative immunoblot of immunoprecipitated Arc oligomers from HFS treated DG. 2 h post-HFS, DG were collected and pooled (from 4 rats) and crosslinked with DSSO (MS cleavable crosslinker) before lysis. Lysates were immunoprecipitated using anti-Arc Nb (ALFA-H11) or anti-Arc antibody. Purified complexes were separated and probed with anti-Arc antibodies to identify immunoreactive bands of interest. **(E)** Densitometric quantification of immunoblots from HFS-LTP. Fold change in immunoprecipitated Arc dimer relative to contralateral DG. Values are expressed as mean ± SEM (*n* = 3). **(F)** Verification of Arc presence in putative Arc dimers and oligomers. Fold change in normalized Arc abundance at ~55, ~130, and ~ 250 kDa in immunopurified samples relative to contralateral DG.

Mass spectrometry of immunoprecipitated Arc (IP–MS/MS) was used to further assess detection of oligomers. First, we ectopically expressed mTq2-Arc under the CMV promoter in HEK293FT cells, which do not express native Arc (Figure 6A). In non-crosslinked samples, we observed a strong monomer band at ~ 75 kDa and a weak band at ~ 150 kDa. The 150 kDa bands correspond to Arc dimers as validated by biophysical analysis of the purified Arc oligomerization motif mutant (Eriksen et al., 2021). Crosslinking increased the 150 kDa band indicating trapping of Arc dimers, while appearance of discrete heavier bands suggests trimers and tetramers (Figure 6B). Arc immunoprecipitation enriched for mTq2-Arc species in DSG crosslinked samples (Figure 6B). Following another round of immunoprecipitation and electrophoresis, CCB stained bands of M_r_ corresponding to monomers, dimers and oligomers were gel-excised and processed for LC–MS/MS (Figure 6A). The MS proteomic analysis identified Arc in the excised bands and absence of known Arc binding partners (Figure 6C; Supplementary Table 2).

Next, we assessed oligomers in HFS-LTP treated DG exposed to the MS-cleavage crosslinker DSSO. Following immunoprecipitation and SDS-PAGE, bands corresponding to ~ 55, ~ 130 and ~ 250 kDa bands were identified by CCB staining and gel-excised for MS analysis (Figure 6D). Consistent with findings in Figures 4C,D, *in situ* DSSO crosslinking preserved Arc dimers and oligomers as seen with DSG crosslinking. A 10-fold increase in the Arc dimer band was detected in ipsilateral DG relative to contralateral control in the input lysate sample (Figure 6E). Arc immunoprecipitation with H11 (ALFA-H11) nanobody or antibody enriched for dimer, which showed fold increases post-HFS of more than 200 fold (Figures 6D,E).

To validate putative dimers and oligomers, we first used MS to confirm the presence of Arc in ~ 130 and ~ 250 kDa bands (Supplementary Table 3). Arc was identified by 15 unique peptides. Anti-Arc antibodies enriched for Arc species with higher efficiency than anti-Arc nanobody. Antibody based immunoprecipitation led to 15, 31 and 6-fold increase (relative to contralateral DG) in normalized Arc abundance at ~ 55, ~ 130 and ~ 250 kDa bands, respectively. The fold increases using anti-Arc nanobody were 4 (~ 55 kDa) and 22 (~ 130 kDa; Figure 6E, left panel). Focusing on ~130 kDa band, we screened MS identified proteins for Arc binding partners and other proteins found in Arc complexes. The Arc band at ~ 130 kDa could be an Arc dimer, Arc interaction with a physiological binding partner, or non-physiological binding forced by crosslinking. Theoretically, protein(s) with a maximum ~ 85 (± 10) kDa M_r_ could associate with Arc to produce the ~130 kDa band. We therefore excluded proteins with M_r_ > 85 kDa, and proteins detected in negative control or IgG samples. We were left with only one protein, Rus family member 1 (Rusf1; C16orf58)—a predicted transmembrane protein of unknown function containing a conserved DUF647 domain (San Diego Supercomputer Center, 2022; Supplementary Table 3). However, identification of Rusf1 is uncertain as only one unique peptide was detected by MS. The protein was not found in the 130 kDa band from the contralateral DG, and it is not a known binding partner or constituent of Arc complexes (Fernández et al., 2017; Nikolaienko et al., 2018). Finally, co-immunoprecipitation confirmed that Rusf1 does not to bind Arc in ipsilateral or contrateral DG tissue post-HFS (Supplementary Figure S4).

## Discussion

This study provides the first evidence of Arc oligomerization in mammalian brain. The results implicate Arc dimers as the predominant low-oligomeric form, exhibiting regional differences in its constitutive expression and pronounced enhanced expression during DG LTP.

Identification of homo-oligomers formed by weak non-covalent interactions is challenging due to disruption of interaction caused by cell lysis and sample processing. To stabilize interactions and facilitate capture of Arc complexes *in situ*, we applied cell permeable crosslinkers to live cell cultures or dissected brain regions. High molecular mass Arc immunoreactive bands on SDS-PAGE could represent Arc self-association, binding to a physiological interaction partner, or non-specific crosslinking. Several observations corroborate detection of oligomers, and particularly Arc dimer formation. (1) Discrete Arc immunoreactive bands of M_r_ corresponding to Arc dimers were detected by two different antibodies raised in different species. Discrete bands suggesting trimers and tetramers were also found. (2) A consistent band pattern was observed in diverse cell types and preparations. This included overexpression of tagged Arc in HEK293FT cells and SH-SY5Y neuroblastoma cells, as well as endogenous Arc expression in neuroblastoma cells, primary cortical neuronal cultures, and adult rat brain regions (DG, CA, and cortex). (3) Crosslinkers with different spacer arm lengths (DSG 7.7 Å, DSSO 10.3 Å, DSP 12 Å) yielded consistent Arc species. (4) DSP-trapped Arc dimers and oligomers were abrogated in reducing sample buffer, confirming the reversibility of chemically-induced crosslinks. (5) MS proteomic analysis of the 130 kDa band obtained following crosslinking and immunoprecipitation confirmed enhanced Arc expression in LTP, with mass spectrometric identification of 15 unique Arc peptide sequences. (6) Absence of known Arc binding proteins or constituents of Arc complexes in the excised 130 kDa band (Fernández et al., 2017; Myrum et al., 2017; Nikolaienko et al., 2018). Only Rusf1 remained as a possible contributor to the 130 kDa band by crosslinking to Arc monomer. Rusf1 is a 426 amino acid protein of unknown function predicted to reside in the ER membrane (San Diego Supercomputer Center, 2022). However, the identification of Rusf1, based on detection of only one unique peptide by MS, is uncertain. Furthermore, co-immunoprecipitation analysis showed that Rusf1 does not interact with Arc. Given recent developments in crosslinking-MS as a tool for structural biology (Piersimoni et al., 2022), a major future goal is to identify the Arc–Arc interaction sites and determine the stoichiometry of low-oligomeric forms.

Several lines of evidence imply a distinct physiological for Arc monomer and low-order monomers that would support rapid actions of activity-induced Arc in synaptic plasticity, for instance interaction with partners involved in actin cytoskeletal regulation and AMPAR trafficking (Eriksen and Bramham, 2022). Studies of bacterially-expressed, purified Arc demonstrate reversible self-association (Byers et al., 2015; Myrum et al., 2015), forming various oligomeric species, including virus-like capsids of 30 nm in diameter estimated to contain about 130 units. Recent work implicates the Arc dimer as the building block for higher-order oligomers (Eriksen et al., 2021). Arc NTD coil-2 is the only region of the protein that self-associates when expressed in isolation. A 7-amino acid stretch, termed the oligomerization motif, is critical for Arc oligomerization above the dimer stage (Eriksen et al., 2021). Although purified mammalian Arc CA does not self-associate, it has conserved dimerization motifs that function in oligomerization of full-length Arc from tetramers to 32-unit oligomers, as shown by dynamic light scattering analysis (Zhang et al., 2015, 2019). It is therefore proposed that dimer-dimer interaction mediated by the NTD enables self-association of CA involved in higher-order oligomerization (Eriksen et al., 2021). A study in knockin mice harboring point mutations in the CA suggests that higher-order Arc oligomers (larger than tetramers) are required for enhanced magnitude LTD, but not for standard Arc-dependent LTD or theta-burst stimulation induced LTP in the hippocampus (Zhang et al., 2019).

Here we provide evidence for endogenous Arc oligomerization following neuronal activity-dependent Arc expression and plasticity. We found enhanced expression of Arc dimer in carbachol treated neuroblastoma cells, BDNF treated cortical neuronal cultures, and following *in vivo* LTP induced by HFS of perforant path input or intrahippocampal infusion of BDNF. This suggests that stimulus-induced Arc rapidly forms dimers. We observed a stable 22-fold increase in dimer during the LTP maintenance phase from 1 to 4 h post-HFS. Previous work showed continuous activation of Arc translation during this period, mediated by persistent BDNF–TrkB signaling (Panja et al., 2014). Infusion of Arc antisense oligodeoxynucleotide during LTP maintenance results in loss of LTP and reduced Arc protein levels within 30 min of infusion (Messaoudi et al., 2007). Thus, Arc protein critical for LTP maintenance has a rapid action and turnover (Zhang and Bramham, 2021). Although Arc is known to undergo proteasomal degradation with a half-life between 30 and 60 min, the stability of oligomeric species is unknown. Continuous production of a metabolically stable dimer would result in its accumulation during LTP maintenance, which we did not observe. Therefore, the dimer is either actively degraded during LTP or assembled into larger oligomers. A rapid degradation would be consistent with a rapid action of Arc in the LTP consolidation phase, for instance through regulation of F-actin dynamics in dendritic spines (Fukazawa et al., 2003; Messaoudi et al., 2007; Nair et al., 2017). With our crosslinking analysis of the 130 kDa band, we are measuring free dimers rather than dimers bound to effectors of intracellular signaling and plasticity. The dimer is of low abundance relative to monomer (ratio of 04–0.6 in DG before and after LTP induction) yet the proportion of dimers may be underestimated as the Arc monomer band in the DSG lane likely includes non-crosslinked protein. At present we are unable to assess high-order Arc homo-oligomers. The heaviest Arc immunoreactive bands at the top of the gel (Figures 3F, 4C; Supplementary Figure S3) could be due to large (mDa) Arc protein interaction complexes of the postsynaptic density as well as homo-oligomers (Fernández et al., 2017). In some LTP experiments, we also found clear dimer detection without DSG crosslinking (HFS-LTP, Figure 3D). It is possible that this detergent-resistant pool reflects a more stable dimer. Methods for visualizing or biochemically isolating native Arc oligomers are needed.

Another salient finding was detection of constitutive Arc dimers in brain. In DG, Arc dimer expression was low under basal conditions, while dimers were prominent in CA and cortex. The regional differences in dimer levels followed the total monomer expression, as measured in non-crosslinked samples. This suggests there are no major differences between regions in the proportion of dimer to monomer (Figure 5). It remains to be seen whether regional differences in dimer levels reflects the proportion of excitatory neurons undergoing Arc transcription, or differences in basal accumulation of dimer.

Several recent studies have provided data on Arc oligomerization based on imaging of ectopically expressed Arc tagged with a fluorescent protein. Fluorescence fluctuation spectroscopy of Arc-EGFP in transfected HeLa cells indicates that Arc is monomeric in the nucleus and monomeric or dimeric in the cytoplasm (Hedde et al., 2022). In a new method termed time-resolved anisotropy with reversibly switchable states (STARSS), a reversibly photo switchable EGFP was inserted between the Arc NTD and linker region (Volpato et al., 2022). These authors report low-copy-number oligomers and larger rigid assemblies in the tens of nanometers in HeLa cells. Using fluorescence fluctuation microscopy and TIRF imaging of transfected SH-SY5Y cells, Goo et al. (2018) observed self-association of Arc-mCherry. At the plasma membrane, Arc transitioned from monomer to dimer at higher concentrations (Goo et al., 2018).

Endogenous expression of Arc in neuroblastoma SH-SY5Y cells has been used to elucidate transcription and translational regulation of Arc. However, the function of Arc in these cells is unknown. The SH-SY5Y cells used in this study are undifferentiated, catecholaminergic neuron-like cell lines that express immature markers including tyrosine hydroxylase and dopamine-β-hydroxylase (Kovalevich et al., 2021). Carbachol stimulation of muscarinic acetylcholine receptors in SH-SY5Y upregulates Arc transcription and translation through activation of extracellular signal-regulated kinase (ERK; Waltereit et al., 2001; Teber et al., 2004; Soulé et al., 2012). The present observation of carbachol-induced Arc dimer formation shows that oligomerization is not restricted to excitatory, glutamatergic neurons with dendritic spines. This makes it interesting to consider non-synaptic, possibly nuclear roles of Arc in SH-SY5Y cells.

In conclusion, a major fraction of endogenous, stimulus-evoked Arc undergoes rapid dimerization. It will be important to elucidate specific functions for dimers as possible mediators of Arc hub signaling in neuronal plasticity and as precursors for higher-order oligomers such as capsids.

## Data availability statement

The mass spectrometry proteomics data have been deposited to the ProteomeXchange Consortium via the PRIDE partner repository with the dataset identifier PXD039612 and 10.6019/PXD039612.

## Ethics statement

The animal study was reviewed and approved by Norwegian National Research Ethics Committee.

## Author contributions

TM and CB designed the research and wrote the article. TM conducted electrophysiological experiments and performed all experiments in SH-SY5Y cell lines, cortical neuronal cultures, and performed most of the biochemical studies. YI provided cortical neurons and supervised biochemical studies. JG performed electrophysiology and some of the biochemical studies. GB performed electrophysiology. TK performed experiments of *in situ* crosslinking of HEK293FT cells. All authors contributed to the article and approved the submitted version.

## Funding

Funding was provided by grants from the Research Council of Norway (249951) and the Trond Mohn Foundation (grant TMS2021TMT04) to CB. TM received a PhD fellowship from the University of Bergen, Medical Faculty. Mass spectrometry-based proteomic analyses were performed by the Proteomics Unit at the University of Bergen (PROBE). This facility is a member of the National Network of Advanced Proteomics Infrastructure (NAPI), which is funded by the Research Council of Norway (INFRASTRUKTUR-program project number: 295910).

## Conflict of interest

The authors declare that the research was conducted in the absence of any commercial or financial relationships that could be construed as a potential conflict of interest.

## Publisher’s note

All claims expressed in this article are solely those of the authors and do not necessarily represent those of their affiliated organizations, or those of the publisher, the editors and the reviewers. Any product that may be evaluated in this article, or claim that may be made by its manufacturer, is not guaranteed or endorsed by the publisher.

## Supplementary material

### SUPPLEMENTARY FIGURE S1

Validation of in-situ chemical crosslinking. SH-SY5Y cells **(A)**, HFS-treated DG **(B)**, and naive untreated DG, CA and Ctx (cortex) **(C)** were DSG crosslinked, lysed, centrifuged and processed for SDS-PAGE. Membranes were immunoblotted with anti-DJ-1 and β-tubulin antibodies. Black arrows show detected monomers and dimers. Detection of endogenous DJ-1 dimers is a positive control and absence of β-tubulin oligomers is a negative control.

### SUPPLEMENTARY FIGURE S2

Arc dimer to monomer ratio in DG after HFS or BDNF-LTP. Arc dimer to monomer ratio was calculated in HFS or BDNF-treated, ipsilateral DG. Values are expressed as mean ± SEM (*n* = 5 for HFS-LTP and *n* = 4 for BDNF-LTP). Mann-Whitney U test.

### SUPPLEMENTARY FIGURE S3

Reversibility of in-situ crosslinking. DSP in-situ crosslinking was tested for reversibility. DSP contains a disulfide bond in its spacer arm, which is cleavable by thiols. Thus, crosslinked samples were denatured in reducing (DTT containing) or non-reducing 2X Laemmli sample buffer before running on SDS-PAGE. Blots were probed against anti-Arc **(A)** and DJ-1 **(B)** antibodies. Black arrows and asterisks show specific and unspecific immunoreactive bands, respectively. # symbol indicates bands corresponding to Arc species larger than dimers.

### SUPPLEMENTARY FIGURE S4

Arc does not interact with Rusf1 protein at baseline or post-HFS. Arc was immunoprecipitated from DG tissue using mouse monoclonal anti-Arc antibody and complexes were separated by SDS-PAGE. Membranes were immunoblotted with rabbit polyclonal anti-Rusf1 antibody. Rusf1 (@ 50 kDa) is detected in the DG input samples, but not in immunoprecipitated samples from ipsilateral or contralateral DG at 1 h post-HFS. Examples from 3 experiments are shown.

### SUPPLEMENTARY TABLE S1

Details of statistical analysis.

### SUPPLEMENTARY TABLE S2

Arc binding partners were not detected at ~150 kDa in ectopic Arc expressing HEK cells. Proteins identified by MS were refined using the following exclusion criteria. 1) exclusion of > 90 kDa proteins for ~150 kDa bands. 2) Exclusion of proteins detected at ~150 kDa in negative (mTq2 vector only transfected HEK293FT cells) samples. 3) Retaining only those proteins detected at ~150 kDa in crosslinked mTq2-Arc transfected HEK293FT cells. 4) exclusion of proteins with a low confidence level. 5) Removal of contaminant proteins.

### SUPPLEMENTARY TABLE S3

Arc binding partners were not detected at ~130 and ~250 kDa. Proteins identified by MS were refined using five exclusion criteria. 1) Removal of > 85 or 205 kDa proteins for ~130 or ~250 kDa bands, respectively. 2) Exclusion of proteins detected at ~130 or ~250 kDa in IgG or NC samples. 3) Retaining only those proteins detected at ~130 or ~250 kDa in both anti-Arc nanobody and antibody immunoprecipitated ipsilateral DG. 4) Exclusion of proteins with a low confidence level. 5) Removal of contaminant proteins.
