# Supplementary figures and images for: Detection of Arc/Arg3.1 oligomers in rat brain: constitutive and synaptic activity-evoked dimer expression in vivo
Source: Front Mol Neurosci. 2023 Jun 9;16:1142361. doi: 10.3389/fnmol.2023.1142361 (PMC10289200; doi:10.3389/fnmol.2023.1142361)

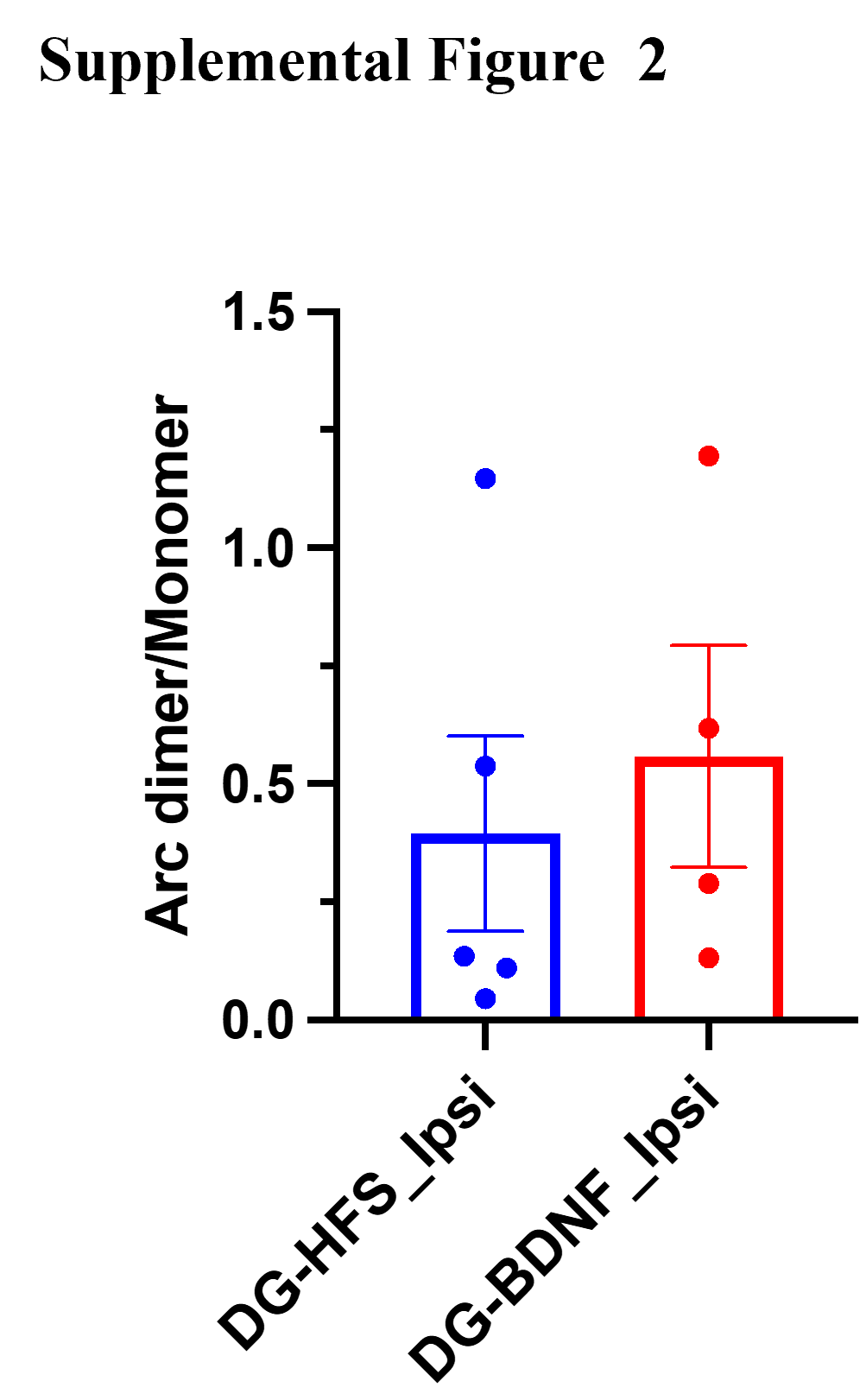

Supplement: Supplementary file 1 [file Image_1.tif]

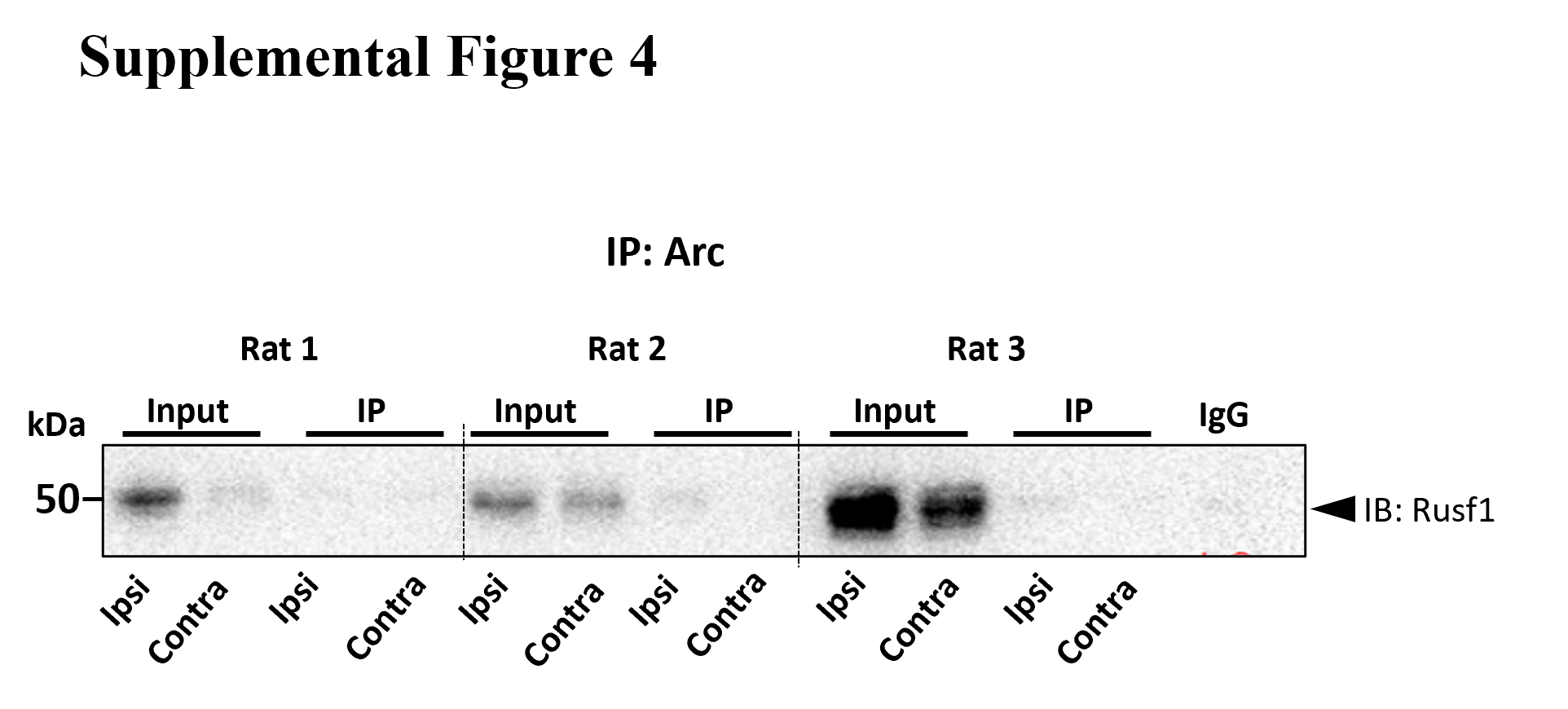

Supplement: Supplementary file 2 [file Image_2.tif]

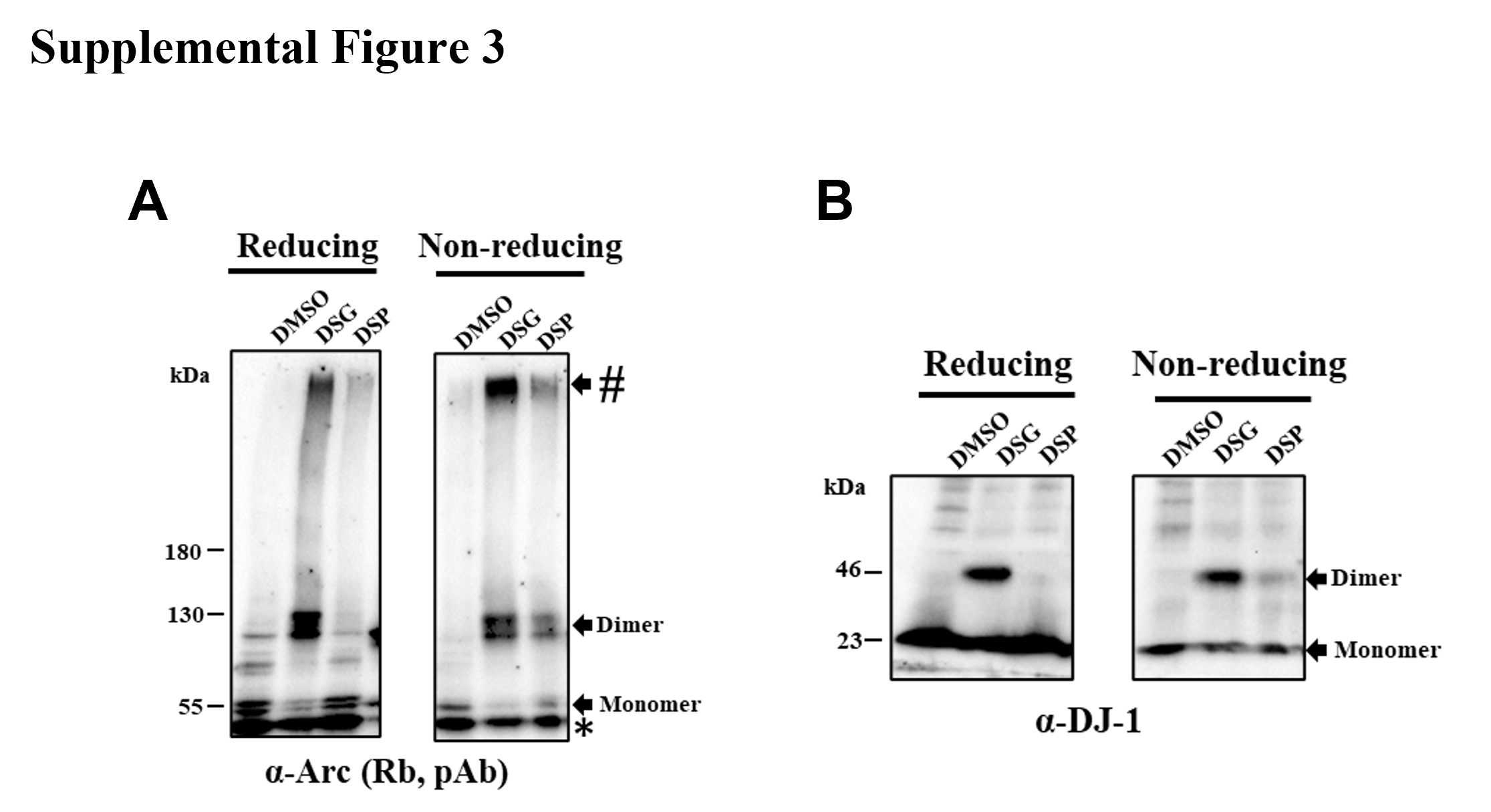

Supplement: Supplementary file 3 [file Image_3.TIF]

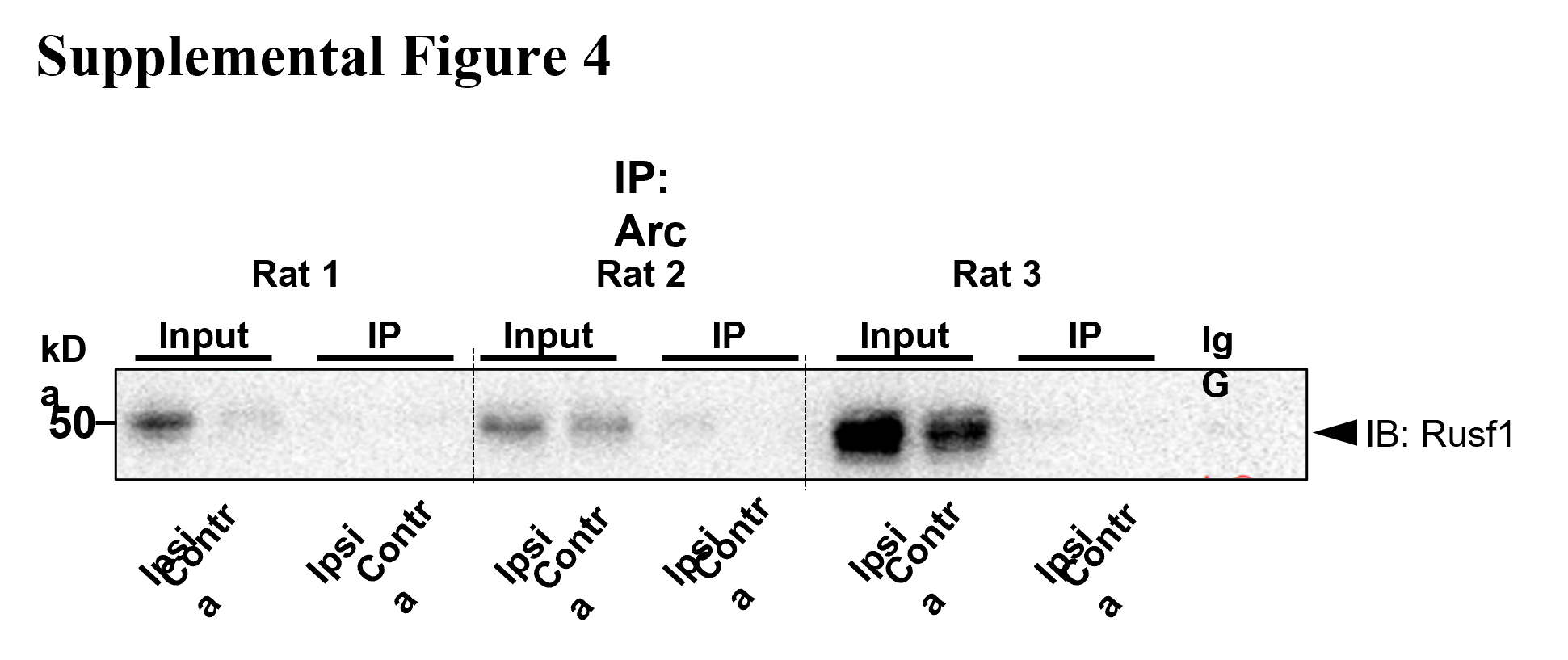

Supplement: Supplementary file 4 [file Image_4.TIF]
