# Supplementary material for: Detection of Arc/Arg3.1 oligomers in rat brain: constitutive and synaptic activity-evoked dimer expression in vivo
Source: Front Mol Neurosci. 2023 Jun 9;16:1142361. doi: 10.3389/fnmol.2023.1142361 (PMC10289200; doi:10.3389/fnmol.2023.1142361)
Supplement: Supplementary file 5 [file Table_1.docx]

**Supplemental Table 1. Statistics details**

| **Sample** | **Groups** | **Statistics used** | **Fig in the manuscript** |
| --- | --- | --- | --- |
| Standard SH-SY5Y cells | Ctrl  vs  Cch60 | Mann-Whitney test  p = 0.0079  Sum of ranks: 15 vs 40  U = 0  Median: 0.08453 vs 2.082  n: 5 vs 5 | Fig. 1C Left |
| Standard SH-SY5Y cells | Ctrl  vs  Cch60 | Mann-Whitney test  p = 0.0079  Sum of ranks: 15 vs 40  U = 0  Median: 1.027 vs 3.808  n: 5 vs 5 | Fig. 1C Right |
| Overexpressing SH-SY5Y cells | Ctrl  vs  Cch5  vs  Cch60 | Kruskal-Wallis test with Dunn’s multiple test  Kruskal-Wallis statistic: 6.767  Number of treatments: 3  Number of values: 15  Ctrl vs Cch60  p = 0.046  Mean rank diff: -7.667  n: 6 vs 3  Cch5 vs Cch60  p = 0.0612  Mean rank diff: -7.3333  n: 6 vs 3  Ctrl vs Cch5  p > 0.9999  Mean rank diff: -0.3333  n: 6 vs 6 | Fig. 1E Left |
| Overexpressing SH-SY5Y cells | Ctrl  vs  Cch5  vs  Cch60 | Kruskal-Wallis test with Dunn’s multiple test  Kruskal-Wallis statistic: 6.900  Number of treatments: 3  Number of values: 15  Ctrl vs Cch60  p = 0.0342  Mean rank diff: -8  n: 6 vs 3  Cch5 vs Cch60  p = 0.0806  Mean rank diff: -7  n: 6 vs 3  Ctrl vs Cch5  p > 0.9999  Mean rank diff: -0.3333  n: 6 vs 6 | Fig. 1E Right |
| Standard and OE SH-SY5Y cells | Ctrl-Std vs  Ctrl-OE | Mann-Whitney test  p = 0.0317  Sum of ranks: 17 vs 38  U = 2  Median: 1.027 vs 5.883  n: 5 vs 5 | Fig. 1G |
| Cortical Neurons | Ctrl  vs  BDNF | Mann-Whitney test  p = 0.1  Sum of ranks: 6 vs 15  U = 0  Median: 0.004351 vs 1.204  n: 3 vs 3 | Fig. 2B Left |
| Cortical Neurons | Ctrl  vs  BDNF | Mann-Whitney test  p = 0.0159  Sum of ranks: 16 vs 39  U = 1  Median: 0.1072 vs 50.09  n: 5 vs 5 | Fig. 2B Right |
| HFS LTP, dentate gyrus (DG) | Contra vs  Ipsi | Mann-Whitney test  p = 0.0357  Sum of ranks: 15 vs 21  U = 0  Median: 0,2173 vs 28.04  n: 5 vs 3 | Fig. 3E Left |
| HFS LTP, dentate gyrus (DG) | Contra vs  Ipsi | Mann-Whitney test  p = 0.0286  Sum of ranks: 10 vs 26  U = 0  Median: 0.6718 vs 18.35  n: 4 vs 4 | Fig. 3E Right |
| BDNF LTP, dentate gyrus (DG) | Contra vs  Ipsi | Mann-Whitney test  p = 0.0286  Sum of ranks: 10 vs 26  U = 0  Median: 0.4909 vs 321.9  n: 4 vs 4 | Fig. 3G Left |
| BDNF LTP, dentate gyrus (DG) | Contra vs  Ipsi | Mann-Whitney test  p = 0.0286  Sum of ranks: 10 vs 26  U = 0  Median: 0.7122 vs 362.4  n: 4 vs 4 | Fig. 3G Right |
| Untreated brain tissues | DG  vs  CA  vs cortex | Kruskal-Wallis test with Dunn’s multiple test  Kruskal-Wallis statistic: 7.848  Number of treatments: 3  Number of values: 11  DG vs CA  p = 0.021  Mean rank diff: -6.833  n: 4 vs 3  DG vs cortex  p = 0.2510  Mean rank diff: -4.5  n: 4 vs 3  CA vs cortex  p = 0.9389  Mean rank diff: 2.33  n: 3 vs 4 | Fig. 5B Left |
| Untreated brain tissues | DG  vs  CA  vs cortex | Kruskal-Wallis test with Dunn’s multiple test  Kruskal-Wallis statistic: 0.8939  Number of treatments: 3  Number of values: 11  DG vs CA  p >0,9999  Mean rank diff: -0.08333  n: 4 vs 3  DG vs cortex  p >0,9999  Mean rank diff: -2.000  n: 4 vs 3  CA vs cortex  p >0,9999  Mean rank diff: -1,917  n: 3 vs 4 | Fig. 5B Right |
| Untreated brain tissues | DG  vs  CA  vs cortex | Row statistics of Arc dimer percentage relative to Arc dimer + monomer  DG: 31.475 ± 8.630, n = 4  CA: 38.386 ± 18.554, n = 3  Cortex: 44.009 ± 5.933, n = 4 | Fig. 5B |
| HFS and BDNF LTP, dentate gyrus (DG) | HFS vs  BDNF | Mann-Whitney test  p = 0.4127  Sum of ranks: 21 vs 24  U = 6  Median: 0.1362 vs 0.4536  n: 5 vs 4 | Supp Fig. 2 |
| HFS and BDNF LTP, dentate gyrus (DG) | HFS vs  BDNF | Row statistics of Arc dimer percentage relative to Arc dimer + monomer  HFS: 22.937 ± 9.240, n = 5  BDNF: 31.669 ± 9.343, n = 4 | Supp Fig. 2 |
| SH-SY5Y cells (Std, OE),  cortical neurons &  dentate gyrus (HFS or BDNF LTP) | Treated samples | Fold change in dimer to fold change in monomer in treated samples relative to control  Kruskal-Wallis test with Dunn’s multiple test  Kruskal-Wallis statistic: 10.12  Number of treatments: 5  Number of values: 22  Cch-Std SH SY5Y vs Cch-OE SH SY5Y  p >0.4209  Mean rank diff: -9.333  n: 6 vs 3  Cch-Std SH SY5Y vs BDNF-Cortical Neurons  p >0.5984  Mean rank diff: -7.400  n: 6 vs 5  Cch-Std SH SY5Y vs HFS-DG-*in vivo*  p >0,9492  Mean rank diff: -7.000  n: 6 vs 4  Cch-Std SH SY5Y vs BDNF-DG-in vivo  p >0.0286  Mean rank diff: -12.50  n: 6 vs 4  Cch-OE SH SY5Y vs BDNF-Cortical Neurons  p >0.9999  Mean rank diff: 1.933  n: 3 vs 5  Cch-OE SH SY5Y vs HFS-DG-in vivo  p >0.9999  Mean rank diff: 2.333  n: 3 vs 4  Cch-OE SH SY5Y vs BDNF-DG-in vivo  p >0.9999  Mean rank diff: -3.167  n: 3 vs 4  BDNF-Cortical Neurons vs HFS-DG-in vivo  p >0.9999  Mean rank diff: 0.4000  n: 5 vs 4  BDNF-Cortical Neurons vs BDNF-DG-in vivo  p >0.9999  Mean rank diff: -5.100  n: 5 vs 4  HFS-DG-in vivo vs BDNF-DG-in vivo  p >0.9999  Mean rank diff: -5.500  n: 4 vs 4 | Supp Fig. 4 |
